# Data Quality and Cost-effectiveness Analyses of Electronic and Paper-Based Interviewer-Administered Public Health Surveys: Systematic Review

**DOI:** 10.2196/21382

**Published:** 2021-01-22

**Authors:** Atinkut Alamirrew Zeleke, Tolga Naziyok, Fleur Fritz, Lara Christianson, Rainer Röhrig

**Affiliations:** 1 Medical Informatics Institute for Community Medicine University Medicine Greifswald Greifswald Germany; 2 Division of Medical Informatics Carl von Ossitetzky University Oldenburg Oldenburg Germany; 3 Institute of Medical Informatics University of Münster Münster Germany; 4 Leibniz Institute for Prevention Research and Epidemiology - BIPS Bremen Germany; 5 Institute for Medical Informatics Medical Faculty of RWTH University Aachen Aachen Germany

**Keywords:** electronic data collection, demographic and health survey, tablet computer, smartphone, mobile phone

## Abstract

**Background:**

A population-level survey (PLS) is an essential and standard method used in public health research that supports the quantification of sociodemographic events, public health policy development, and intervention designs. Data collection mechanisms in PLS seem to be a significant determinant in avoiding mistakes. Using electronic devices such as smartphones and tablet computers improves the quality and cost-effectiveness of public health surveys. However, there is a lack of systematic evidence to show the potential impact of electronic data collection tools on data quality and cost reduction in interviewer-administered surveys compared with the standard paper-based data collection system.

**Objective:**

This systematic review aims to evaluate the impact of the interviewer-administered electronic data collection methods on data quality and cost reduction in PLS compared with traditional methods.

**Methods:**

We conducted a systematic search of MEDLINE, CINAHL, PsycINFO, the Web of Science, EconLit, Cochrane CENTRAL, and CDSR to identify relevant studies from 2008 to 2018. We included randomized and nonrandomized studies that examined data quality and cost reduction outcomes, as well as usability, user experience, and usage parameters. In total, 2 independent authors screened the title and abstract, and extracted data from selected papers. A third author mediated any disagreements. The review authors used EndNote for deduplication and Rayyan for screening.

**Results:**

Our search produced 3817 papers. After deduplication, we screened 2533 papers, and 14 fulfilled the inclusion criteria. None of the studies were randomized controlled trials; most had a quasi-experimental design, for example, comparative experimental evaluation studies nested on other ongoing cross-sectional surveys. A total of 4 comparative evaluations, 2 pre-post intervention comparative evaluations, 2 retrospective comparative evaluations, and 4 one-arm noncomparative studies were included. Meta-analysis was not possible because of the heterogeneity in study designs, types, study settings, and level of outcome measurements. Individual paper synthesis showed that electronic data collection systems provided good quality data and delivered faster compared with paper-based data collection systems. Only 2 studies linked cost and data quality outcomes to describe the cost-effectiveness of electronic data collection systems. Field data collectors reported that an electronic data collection system was a feasible, acceptable, and preferable tool for their work. Onsite data error prevention, fast data submission, and easy-to-handle devices were the comparative advantages offered by electronic data collection systems. Challenges during implementation included technical difficulties, accidental data loss, device theft, security concerns, power surges, and internet connection problems.

**Conclusions:**

Although evidence exists of the comparative advantages of electronic data collection compared with paper-based methods, the included studies were not methodologically rigorous enough to combine. More rigorous studies are needed to compare paper and electronic data collection systems in public health surveys considering data quality, work efficiency, and cost reduction.

**International Registered Report Identifier (IRRID):**

RR2-10.2196/10678

## Introduction

Until well-established civil and vital statistics systems are in place in low- and middle-income countries (LMIC), monitoring sociodemographic events using data on vital societal statistics will remain dependent on alternative data sources. Public health surveys—such as censuses, demographic and health surveys (DHS), and health and demographic surveillance—serve as a data lifeline for these countries [1,2]. Mortality and morbidity indicators, service utilization, and population-level program impact evaluations are usually calculated from household-level data. Further analysis of these population-level epidemiologic indicators is helpful in identifying the determinants of mortality and morbidity. Data collection and management is the first step in the process of evidence generation from household surveys, in which data quality errors could be introduced or prevented. Avoiding errors at this stage is the first-line choice to avoid inherited errors in further data management processes [1,3,4].

The current classical data collection and management processes in LMIC are heavily dependent on paper-based manual methods [4,5]. Paper-based data collection requires extensive human and material resources, especially for large-scale surveys. It also incurs high printing and data entry costs and requires extra data quality assurance steps during and after data collection. Moreover, it takes a long time for an error-free data set to be ready for analysis [6,7]. The intrinsic mode of paper-based data collection affects the data quality, timeliness, and cost of survey implementation, among other factors [8-10].

The rapid development of the global telecommunications infrastructure provides an opportunity for mobile and wireless technologies (mobile health [mHealth]) to support health services and research. Harnessing this technology’s potential, particularly in LMIC where the disease burden is highest, is becoming a popular strategy led by relevant activities in World Health Organization member countries [11]. There are diverse mHealth solutions broadly categorized as a tool to support communication between health service institutions and individuals. These include health call centers; reminders to attend appointments; providing access to information and education for health care professionals, for example, access to electronic health care databases and clinical decision support systems; and supporting health monitoring and surveillance (eg, data collection and reporting in health surveys, surveillance, and patient monitoring) [12].

The implementation of tablet- or smartphone-based data collection tools is becoming increasingly popular in public health surveys to mitigate challenges encountered in paper-based data collection [13,14]. Compared with face-to-face interviews, a self-administering mode of electronic data collection tools could potentially increase the response rate among stigmatized groups. These tools have been tested in the contexts of drug abuse [15] and sexual health or HIV [16-18] in public health. The findings conclude that respondents prefer electronic data collection tools as a solution for reporting sensitive information.

Considering data collection in clinical trials, electronic clinical report forms (eCRF) show a potential advantage over paper-based clinical case report forms (CRF) [19-22]. Studies have identified the relative advantages of electronic data capturing tools in terms of data quality, timeliness, and implementation cost.

A handful of experience reports are available on the use of electronic data collection methods in health and demographic surveillance systems (HDSS) in the International Network for the Demographic Evaluation of Populations and Their Health (INDEPTH) network. The HDSS site in Malawi used an OpenHDS data system as a means of GPS data collection [23]. One surveillance site in Kenya also reported the adoption of technological innovation using OpenHDS to manage a large-scale malaria survey in western Kenya. The findings asserted that electronic data collection (EDC) enabled the collection of demographic and malaria data quickly and effectively. Moreover, the possibility of real-time data quality controls using the system led to an efficient workflow and subsequent cost savings [24]. The Kombewa HDSS in Kenya also collected data electronically using PDAs and computer notebooks [25,26]. Since 2010, the Magu HDSS site in Tanzania has used EDC to enable enumerators to record household information directly in the PDA [27]. The Dabat HDSS site in northwest Ethiopia also reported the use of PDAs as a means of data collection [6,28]. Most HDSS and DHS still use a paper-based data collection system, and those sites with EDC implementation experience have rarely published their experience or the comparative impact of EDC and paper-based data collections. Despite the individual implementation experiences that suggest that EDC tools can improve data quality and work efficiency and reduce overall survey costs, systematic reviews of the available evidence are limited. The focus of the available systematic reviews is primarily on the mixed potential of mHealth, not specifically on the impact of mobile devices on improving the data collection and management processes in surveys [13,29,30]. Therefore, the impacts of EDC tools in surveys need to be separately analyzed and reported. The available Cochrane systematic review on the impact of data quality parameters focuses on self-administered EDC tools and excludes interviewer-administered methods [14]. In the case of face-to-face interviews, the data collection process involves interaction between the questionnaire, respondent, and interviewer. The difference in the mode of questionnaire administration can have serious effects on data quality [9]. Moreover, conducting face-to-face surveys has more organizational costs involved than self-administered surveys.

Therefore, a systematic review that considers interviewer-administered data collection may complement this evidence. We found no systematic review that analyzed the data quality and cost-effectiveness of electronic and paper-based interview-administered public health surveys. The objective of this systematic review is to synthesize the evidence on the effect of using EDC systems on data quality and cost reduction in public health surveys, with a focus on studies reporting comparative impacts of paper-based data collection and EDC. 

## Methods

We registered a detailed protocol with PROSPERO, an international database of prospectively registered systematic reviews, with the registration number CRD42018092259. PRISMA (Preferred Reporting Items for Systematic Review and Meta-Analysis) guidelines were used to report our systematic review [31,32]. The protocol of this study has been published [33].

### Inclusion Criteria

We assessed studies that investigated the effect of EDC methods on improving the data quality and cost-effectiveness in public health surveys or surveillance, compared with traditional paper-based data collection methods. We included all mobile apps with technologies that directly support the data collection process by enabling data collectors and interviewers to collect and send data as well as enabling supervisors and data managers to monitor the data collection process. The study participants included in our review are defined as data collection tool users who use a method of data collection.

Studies with the following characteristics were included:

- The study compared either data quality or cost-effectiveness or both as primary outcomes and reported these in the paper.
- The intervention consisted of mobile information and communication technology devices along with mobile apps, which include PDAs, cellphones, smartphones, and tablet computers—devices used specifically for data collection and reporting processes during surveys.
- The control and intervention groups were compared in face-to-face interview-administered surveys conducted at the household level.
- Demographic surveillance sites were based on clinical settings and not mandated for standard clinical trials (eg, CRF vs eCRF).
- The paper was published between January 2008 and December 2018.

### Search Information Source and Search Strategies

Studies were identified through systematic searching in the following electronic databases: MEDLINE via Ovid, CINAHL via EBSCO, PsycINFO via Ovid, EconLit via EBSCO, the Social Science Citation Index, the Science Citation Index via the Web of Science and CENTRAL, and the Cochrane Library (Table 1). In addition, the reference lists of all the included citations were screened. We also searched clinical trial registries for unpublished and in-progress studies.

**Table 1:** Subject term translations for individual databases

MEDLINE and Cochrane	PsycINFO	CINAHL
Mobile applications	Not available	Mobile applications
Computers, handheld	Mobile devices, computer peripheral devices	Computers, handheld
Electronic health records	Not available	Electronic health records
Cell phone	Cellular phones	Cell phone
Surveys and questionnaires	Surveys; questionnaires	Data collection methods
Interviews as topic	Interviews	Included in data collection methods
Costs and cost analysis	Costs and cost analysis	Costs and cost analysis
Data accuracy	Not available	Not available

The search strategy reported in the protocol was refined and updated in collaboration with a research librarian. This strategy considered 3 categories: the technology or intervention used (eg, mobile device, mobile phone, mHealth, or EDC), area of application (eg, data collection, demographic and health survey, or large-scale survey), and the outcome of interest (eg, data quality, missing data, and cost-effectiveness). We linked synonyms and controlled vocabulary with Boolean operators *OR* and the categories with the operator *AND*. Textbox 1 presents the search strategy in MEDLINE via Ovid. Appropriate modifications to control for vocabulary and syntax were made to the search strategy for each database (Textbox 1). Additional search strategies for PsycINFO, CINAHL, Web of Science, and Cochrane databases are presented in the supplementary file (Multimedia Appendix 1). All searches were conducted in January 2019.

**Search strategy in MEDLINE via Ovid.**

MEDLINE(R) and Epub Ahead of Print, via Ovid

1. (((tablet or handheld or hand held or electronic) adj2 (device* or computer*)) or ((electronic or digital) adj2 (form? or data capture* or survey* or case report form? or data collection?)) or Open Data Kit or ODK or EDC or eCRF or eHealth or mHealth or digital health or Android or tablet? or PDA? or personal digital assistant? or app? or (mobile adj2 (technolog* or application? or app?)) or ((mobile or cell* or smart) adj2 phone*) or smartphone* or cellphone*).ti,ab.

2. exp “mobile applications”/

3. exp ”computers, handheld”/

4. exp “electronic health records”/

5. exp “cell phone”/

6. or/2-5

7. 1 or 6

8. (field work or fieldwork or HDSS or CAPI or computer assisted personal interviewing or questionnaire* or survey* or interview* or (population adj2 surveillance) or DHS or EDC or (data adj2 (gather* or captur*)) or (health and demographic surveillance system?)).ti,ab.

9. exp “surveys and questionnaires”/

10. exp “interviews as topic”/

11. or/9-10

12. 8 or 11

13. ((cost? adj2 (analy?s or comparison* or saving? or measure? or effectiv* or reduction? or reduce? or reduction or reducing or decrease? or decreasing)) or (cost benefit adj2 (analy?s or comparison* or measure?)) or (cost utility adj2 (analy?s or comparison* or measure?)) or economic evaluation? or quality control? or (data adj2 (quality or accuracy or accurate* or error? or error rate? or incomplete* or complete* or inaccurate* or inaccuracy or valid*))).ti,ab.

14. exp “costs and cost analysis”/

15. exp “data accuracy”/

16. or/14-15

17. 13 or 16

18. 7 and 12 and 17

19. Limit 18 to yr=“2008 -Current”

### Study Selection

We imported all citations from all databases to EndNote for deduplication management and further screening. Although we planned to use the Covidence web-based screening tool to manage the title and abstract screening process, we finally chose the Rayyan QCRI (Qatar Computing Research Institute) screening tool because it is freely available and provides sufficient screening functionalities. Two authors (AZ, MPH in Health Informatics, and TN, MSc in Informatics) independently screened the titles, abstracts, and full text, based on the inclusion criteria. Disagreements and uncertainty on the screening results were first resolved through discussion among the reviewers, followed by consultation with the third (FF, Postdoc in Medical Informatics) and fifth authors (RR, Professor in Medical Informatics). We used a pretested and standardized (through calibration exercise) Microsoft Excel sheet for data extraction based on the inclusion criteria and the objectives of the review.

### Data Management and Extraction Process

Two reviewers extracted the following information from the papers:

- Bibliographic information (authors, titles, journals, and year of publication).
- Characteristics of the intervention (eg, hardware, software, and networking).
- Study methods (design, setting, participants, and sample size).
- Assessed outcomes (data quality, cost-effectiveness, and others).
- Quantitative or qualitative summary of the main findings, including descriptive frequencies and statistical tests.

The full description of the data extraction items can be accessed in the published protocol [33].

### Risk of Bias or Quality Assessment

Randomized controlled trials are suitable for evaluating whether drugs are effective; however, for interventions that involve health care delivery modes, it may not be appropriate or possible to conduct a randomized controlled trial. We aimed to assess the quality of the data in the included studies using parameters such as random sequence generation, allocation concealment, blinding of participants and personnel, blinding of outcome assessment, incomplete outcome data, selective reporting, and other biases. However, the included studies were neither randomized controlled trials nor nonrandomized trials with clinical outcomes; they were mainly prospective comparative experimental studies, cross-sectional studies, or historical secondary data record comparisons. The remaining studies were a one-time feasibility study or experience reports from the implementation or use of an EDC tool in public health practice.

### Data Synthesis

There was substantial heterogeneity among the studies concerning the intervention (mobile electronic data capturing tools such as PDA, smartphone, or tablet computer and the app they used), outcome types (error rate and missing or inaccurate data), and level of outcome measurements (sample level, household level, and variable level) of the mHealth interventions and study outcomes.

The studies were found to be noncombinable, and combining these studies would not have been methodologically sound. Consequently, we performed a narrative synthesis of the studies. 

## Results

### Study Selection and Characteristics

**Figure 1:**
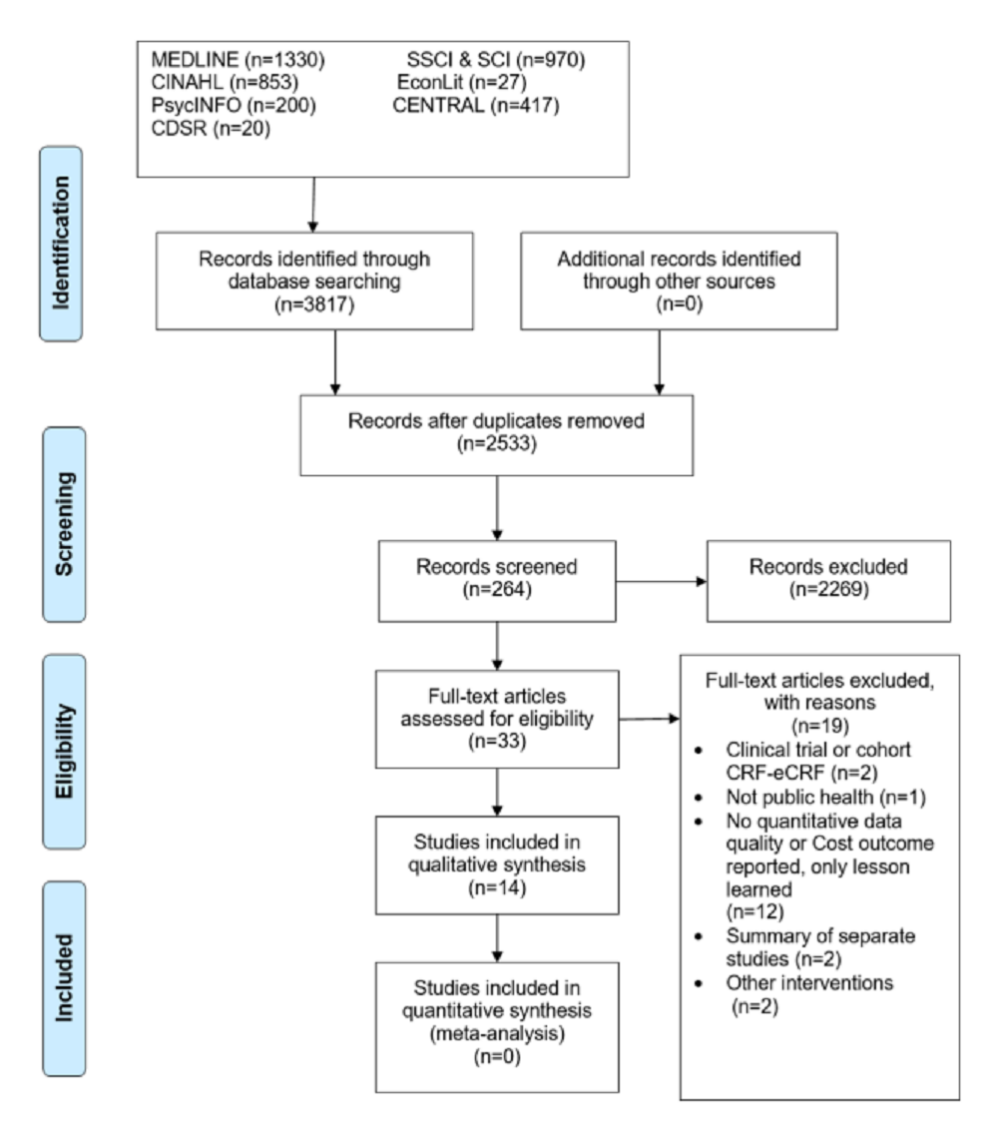
Screening process based on the PRISMA (Preferred Reporting Items for Systematic Review and Meta-Analysis) flowchart template.

The search performed in the included databases yielded 3817 results. After deduplication, 2533 results were exported to the Rayyan QCRI screening tool (Figure 1). Of these, 2500 papers were discarded after title and abstract screening, as these papers clearly did not meet our criteria. Of the 33 full-text papers included, only 14 met the amended inclusion criteria. The original protocol was aimed at including comparative studies that addressed paper-based and electronic tools in the same study, conducted household-level data collection in a community field setting, and reported primary outcomes (data quality or cost-effectiveness) of both data collection tools in the same paper. Only 7 studies (that are heterogeneous) fulfilled these criteria. Due to the limited evidence, we extended our inclusion criteria to cover studies that use the tools in demographic surveys or surveillance systems in a clinical or hospital setting. We also included one-sided study design papers that only reported primary outcomes (cost or data quality) and EDC methods, without formal comparison with paper-based data collection methods. This widening of the scope provided an additional 7 papers (3 comparative and 4 noncomparative EDC papers) to bring the total to 14: 10 comparative and 4 noncomparative single-arm studies were included for final full-text extraction (Figure 1).

### Study Characteristics

The final systematic synthesis analysis included 14 studies conducted in 12 LMIC. Of these 14, 10 [7,34-42] comparatively assessed the outcomes using paper-based data collection and EDC in the same analysis. Furthermore, 4 papers [43-46] reported either data quality or cost-related outcomes or both in a study conducted using an EDC tool (Table 2).

**Table 2:** Study and content characteristics of the included papers

Category		Studies, n	References
**Country (n=14)**			
	Kenya	2	[36,45]
	Ethiopia	1	[7]
	China	2	[42,43]
	Malawi	1	[39]
	India	1	[37]
	Philippines/Bangladesh	1	[34]
	Sudan	1	[35]
	Burkina Faso	2	[40,44]
	Tanzania	1	[38]
	South Africa	1	[41]
	Nigeria	1	[46]
**Study setting (n=10)**			
	Household community setting	7	[7,34,35,37-40]
	Clinical/hospital setting	3	[36,41,42]
**Comparison of paper-based and electronic data collection (n=10)**			
	Both from the same study	8	[34,35,37-42]
	Compared from 2 studies conducted at different times	2	[7,36]
**Purpose of the study design (n=7)**			
	Primarily designed to evaluate paper-based and electronic tools	5	[35,37,39,41,42]
	Secondary byproduct of another primary survey	2	[7,36]
**Types of outcomes (n=10)**			
	Only data quality outcomes	1	[35]
	Only cost outcomes	2	[34,40]
	Both cost and data quality outcomes	7	[7,36-39,41,42]
**Level of data quality outcome assessment (n=8)**			
	Household level	2	[7,38]
	Questionnaire level	4	[7,35,41,42]
	Variable level	3	[36,37,39]
**Type of data quality outcome comparison (n=8)**			
	Missing	4	[7,36,38,39]
	Inaccurate	4	[7,36,38,39]
	Mixed (identified as error)	5	[35,37,38,41,42]
**Economic evaluation type (n=11)**			
	Complete input cost	3	[36,38,40]
	Partial (differential) cost	8	[7,37,39,41-43,45,46]
**Usability/user preference evaluation (n=14)**			
	Reported after formal evaluation	6	[7,34,37-39,42]
	Reported with informal discussion	1	[36]
	No user evaluation information	7	[35,40,41,43-46]
**Study, intervention, or evaluation year (n=10)**			
	2008-2012	5	[36,38,41,42,44]
	2013-2018	5	[34,35,37,43,46]
**Publication year (n=14)**			
	2008-2012	2	[42,46]
	2013-2018	12	[7,34-41,43,45,46]

Furthermore, 5 studies [35,37,39,41,42] were primarily intended to evaluate and compare data quality and cost-related outcomes from a prospective study design using paper-based and electronic tools. The remaining 5 papers [7,34,36,38,40] reported the outcomes from previous technology utilization experiences. The reported outcomes were not primarily intended to evaluate the tools; rather, data quality and cost-related outcomes were extracted from surveys at different times.

Regarding the settings, 7 studies [7,34,35,37-40] in the comparative group included data from a household-level survey, and 3 studies [36,41,42] conducted surveys in clinical settings or research centers.

### Types of Outcomes

#### Data Quality Outcomes

**Figure 2:**
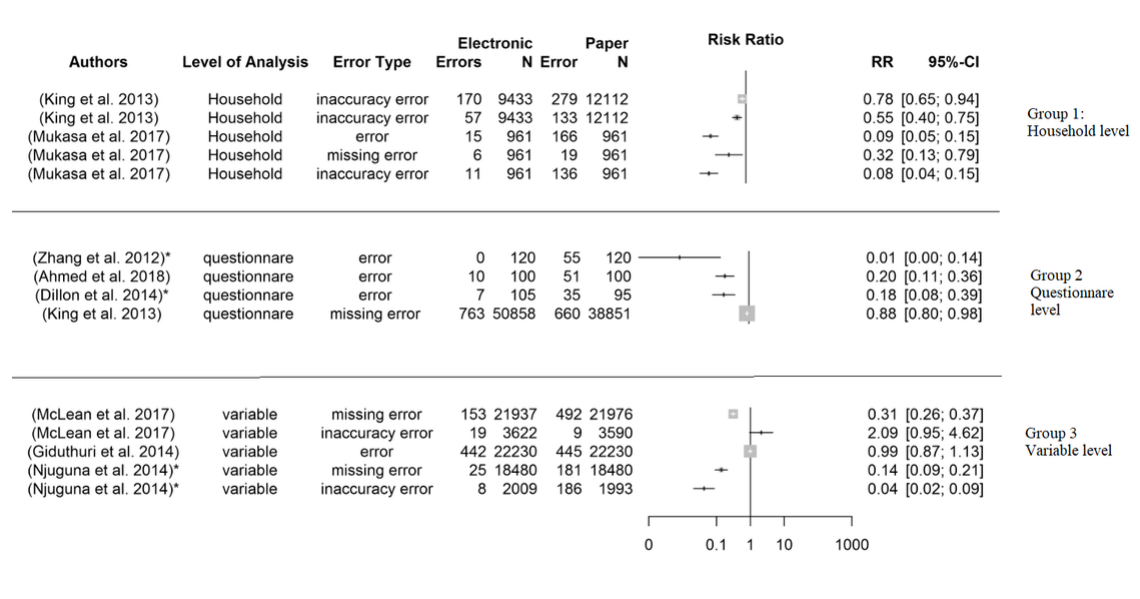
Forest plot comparison of heterogeneity characteristics of the data quality outcomes.

Data quality outcomes, as defined in the methods section, comprise the frequency of errors (incomplete, missing, or inaccurate items) on 3 levels, based on the reported outcomes at the household, questionnaire, and variable levels. At the household level, the incidence of 1 or more errors among the total number of households included in the surveys and data analysis were reported in 2 papers [7,38]. Similarly, the frequency of 1 or more errors per complete questionnaire, regarded as a questionnaire-level error, was reported in 4 studies [7,35,41,42]. At a variable level, a count of the errors in a complete set of questions or variables in questionnaires measured as a variable error were mentioned in 3 papers (Figure 2) [36,37,39].

The cost of implementing electronic and paper-based data collection processes was estimated by most studies (12 out of 14). The majority of these estimated the partial or differential costs unique to that study and its EDC [7,37,39,41-43,45,46]. Only 3 papers listed the full implementation cost [36,38,40] for both the study and the EDC. Except 2 studies that compared costs per correctly entered data observation or error-free databases [39,41], none attempted to link the data quality outcome measures to the cost inputs.

#### Effect on Data Quality: Missing Data and Inaccuracy Errors

Errors are reported in terms of missing and inaccurate data in 4 studies [7,36,38,39], while a combination of both errors as a single error indicator is reported in 4 others [35,37,38,42] (Table 3).

**Table 3:** Extracted data quality outcomes.

Study and country	Type of comparison, study design, and setting^a^	Study population and sample unit	Intervention: device or app	Methods of paper-based and electronic tool administration	Outcome measurement characteristics^b^	Result	Biases (selection, information, or confounding)
Ahmed et al [35], Sudan	B: Exploratory pilot study nested and experimented in cross-sectional household surveys	Sample unit: respondents Sample size: 100 for each (PPDC^c^ and EDC^d^)	EDC: Smartphone ODK^e^ app	Daily sample randomization for EDC or PPDC. 1 respondent simultaneously interviewed by 2 interviewers with different tools (PPDC and EDC)	A: Data quality errors, questions with no answers, or wrong use of the skip pattern B: After data entry C: Errors measured at the questionnaire level D: Used similar questionnaires (BOLD^f^ survey core questionnaire)	PPDC: 51 errors from 100 questionnaires EDC: 10 errors from 100 electronic forms 83% errors in PPDC	Selection and information
King et al [7], Ethiopia	A: Pre- and postdesign in full EDC implementation evaluation in cross-sectional household surveys	Sample units: Households PPDC: 9433 EDC: 12,112 Individuals enumerated in PPDC: 38,851 and in EDC: 50,858 Individuals examined in PPDC: 33,800 and in EDC: 38,652	EDC: For example, tablet computer or self-developed Android app	Paper and electronic surveys at different places and times, 1 data collector at a time	A: Data quality, percentage of individuals enumerated with at least 1 blank field in the census record Percentage of households with incorrect unique identifying number (inaccuracy at the household level) GPS with blank entries (missing at the household level) The proportion of total time B: After data entry (raw data sets) C: At the questionnaire and household levels D: Used the same questionnaire (Trachoma Impact Evaluation Survey questionnaire)	PPDC: Missing rate at the questionnaire level is 1.7% EDC: Missing 1.5% at P=.01 PPDC: Missing rate in GPS data 0.6% (N=9263) EDC: Missing rate in GPS 1.1% (N=12,064; P<.01; person-days) PPDC: Proportion of total time 790 person-days EDC: Proportion of total time 511 person-days	Selection, information, and confounding
McLean et al [39], Malawi	A: Prospective evaluation nested and experimented in cross-sectional household surveys at HDSS^g^ sites	PPDC: 426 respondents EDC: 558 respondents Time: 3 weeks	EDC: Tablet computer and smartphone, ODK app	Independent parallel EDC or PPDC, 1 data collector at a time	A: Missing data defined as not asked (blank; discounting *not applicable* blank questions) or as blank and entered as unknown combined, Internal validity: defined as a field with an impossible or inconsistent value and time for submission B: Not clear C: Variable-level error count D: Used different questionnaires	PPDC: Missing 492 (2.2%) of 21,976 fields EDC: Missing 153 (0.7%) of 21,937 fields (RR^h^ 3.2, 95% CI 2.7-3.8) PPDC: Internal inconsistencies in 19 (0.5%) of 3590 fields EDC: Internal inconsistencies in 9 (0.2%) of 3622 fields (RR 0.5, 95% CI 0.2-1.1) Time from interview to data availability on the database PPDC: mean 3.4 days (range 3.0-3.7) EDC: mean 2.1 days (range 2.0-2.3)	Selection and information
Giduthuri et al [37], India	A: Prospective experimental comparative study designed for households selected randomly	98 interviews in EDC and PPDC	EDC: Tablet computer/ODK app	A respondent simultaneously interviewed by 2 interviewers with different tools (PPDC and EDC)	A: Discrepancies with reference to device-attributable errors Paper entries incorrect or missing and tablet entries missing because paper interviewer (lead) did not follow the skip logic Tablet entries incorrect or missing, paper entries missing because tablet interviewer’s (lead) skip logic was considered an error of EDC B: During data entry C: Variable-level error count D: Used the same questionnaire	PPDC: Mean number of paper-attributable errors 4.68 (445/22,230, 2.01%) EDC: Mean number of tablet-attributable errors 4.65 (442/22,230, 1.99%)	Selection, information, and confounding
Mukasa et al [38], Tanzania	A: Retrospective record review of household survey data	961 households for EDC and PPDC	EDC or PDA BlackBerry customized HRS^i^	Repeated survey, PPDC first, followed by EDC, 1 data collector at a time	A: Error category from the database: accuracy, logic, and consistency; range; and completeness and missing values B: After data entry from database C: Error identified at the household level D: Used the same questionnaire	Households with errors PPDC: 166 (17%) EDC: 15 (2%) PPDC more likely with accuracy errors (79%; 95% CI 72%-86%); EDC: (58%; 95% CI 29%-87%) Errors in EDC more likely to be related to completeness (32%; 95% CI 12%-56%) than in PPDC (11%; 95% CI 7%-17%)	Selection, information, and confounding
Zhang et al [42], China	B: Prospective comparison study conducted in a clinic	60 mothers for each tool (EDC and PPDC)	EDC: smartphones, leased software	A respondent simultaneously interviewed by 2 interviewers with same tools (PPDC or EDC). Random assignation of respondents to one of the tools	A: Data quality errors; missing confirmation of default option, survey date, missed questions, 2 options circled, wrong options chosen, date, ID; database completion (data entry, checking, and data cleaning) B: Before and after data entry C: Questionnaire-level error count D: Used the same questionnaire	Households with errors PPDC: 55/120 EDC: 0 Questionnaire pairs with no recording variations EDC: 15/60 PPDC: 20/60 EDC: 134 of 186 variables (72.0%) did not have any recording variation PPDC: 126 of 184 variables (68.5%) did not have any recording variation In data entry: 65.0% (78/120) of the PPDC records did not match database completion time PPDC: 16 hours; EDC: 30 min	Selection, information, and confounding
Njuguna et al [36], Kenya	A: Pre- and postimplementation evaluation in hospital-based surveillance data collection	EDC: 880 questionnaires (May 2011-June 2012) PPDC: 880 questionnaires (January 2010-June 2011)	EDC: smartphones, FAST^j^ app	Paper and electronic surveys at the same place but different times, 1 data collector at a time	A: Incomplete records or variables in selected questions in a questionnaire; questions requiring responses with or without programmed checks on the smartphone version; percentage of erroneous and inconsistent responses in the questionnaires B: Error count from original responses in the paper C: Variable-level error count D: Used the same questionnaire	Missing error: EDC: 25/18,480 PPDC: 181/18,299 Inaccuracy error: EDC: 8/2009 PPDC: 186/1807	Selection, information, and confounding
Dillon et al [14], South Africa	B: Prospective data collection in case control study in a hospital setting	EDC: 105 respondents PPDC: 95 respondents	EDC: tablet computer, self-developed app	One data collector interviewed all respondents either with paper or with electronic tools in a random order	A: Data collection errors defined as nonsensical or impossible inputs, missing data, or inputs inconsistent or incompatible with previous responses during the interview; minor errors classified as differences of 1 year or less in date calculations. Major errors classified as all other error types B: Not clear C: Variable-level error count D: Used similar questionnaires	Overall number of errors per 100 questions EDC: 0.17 errors; PPDC: 0.73 errors P<.001 Interview duration (EDC: 5.4 min; PPDC: 5.6 min)	Selection, information, and confounding

^a^A: full implementation; B: pilot testing.

^b^A: type of outcome; B: stage of error assessment for paper questionnaire; C: error measurement level; D: questionnaire name/s similarities.

^c^PPDC: pen and paper data collection.

^d^EDC: electronic data collection.

^e^ODK: Open Data Kit.

^f^BOLD: Burden of Obstructive Lung Disease.

^g^HDSS: health and demographic surveillance systems.

^h^RR: risk ratio.

^i^HRS: Household Registration System.

^j^FAST: Field Adapted Survey Toolkit.

An exploratory pilot study nested and experimented in a cross-sectional household survey in Sudan compared data quality errors, questions with no answers, or incorrect use of the skip pattern in 100 convenience samples. A pair of data collectors simultaneously interviewed each respondent—1 with pen and paper and 1 with electronic tools—and recorded the data separately. In the paper-based data collection, 51 of the 100 questions had one or more errors, compared with 10 errors in the electronically submitted forms [35].

A study in India by Giduthuri et al [37] also compared error rates between paper-based and electronically collected data from a comparative prospective experimental study. The data collectors were randomly assigned to use either pen and paper or EDC tools while interviewing each respondent simultaneously. Audio-recorded data during the survey were used as a reference to compare discrepancies and device-attributable errors. According to the reference, paper errors indicate when a paper entry was incorrect and when a tablet entry was missing because the paper-based tool interviewer (lead) did not follow the skip logic. Furthermore, tablet entries were incorrect or missing and paper entries were missing because of the electronic tool interviewer’s (lead) skip logic was considered an error of the electronic data collection tool. The mean number of paper-attributable errors was 4.68 (445/22,230, 2.01%), while the mean number of tablet-attributable errors was 4.65 (442/22,230, 1.99%); thus, no significant differences were observed [37].

A study in China compared smartphone and paper-based data collection in an infant feeding practice survey conducted in rural clinics. Purposive sampling techniques were used to select 120 mothers, 60 per survey tool group. Two data collectors with the same tool (paper or electronic) interviewed 60 mothers in random order, yielding 120 records for each tool. For the paper-based questionnaire, 55 of 120 questionnaires had 1 or more errors or missing data. The most frequent error was a missing confirmation of the default option, which was observed 156 times in 49 questionnaires. No missing error was reported for the EDC tool group [42]. The mean duration of an interview was 10.22 (SD 2.17) min for the smartphone method and 10.83 (SD 2.94) min for the pen and paper method. Moreover, database completion took 16 hours (including data entry, checking, and data cleaning) for pen and paper data collection (PPDC), while it took half an hour for EDC [42].

A prospective evaluation experiment conducted a nested, ongoing, cross-sectional household survey at HDSS sites in Malawi. In 3 weeks, 426 interviews with PPDC and 558 interviews with EDC were conducted. Data collectors independently interviewed different households in a *1 data collector in 1 home* mode. Missing data were defined as not asked (blank; discounting *not applicable* blank questions) or as blank and entered as unknown combined. Internal validity was defined as a field with an impossible or inconsistent value and time for submission. In paper questionnaires, missing data were reported in 492 (2.2%) of 21,976 fields, compared with 153 (0.7%) of 21,937 fields in electronic forms (risk ratio [RR] 3.2, 95% CI 2.7-3.8). Internal inconsistencies were found in 19 (0.5%) of 3590 fields collected by PPDC compared to 9 (0.2%) of 3622 fields for EDC (RR 0.5, 95% CI 0.2-1.1) [39]. Moreover, the mean data availability duration in databases was 3.4 days (95% CI 3.0-3.7) in PPDC compared with 2.1 days (95% CI 2.0-2.3) for EDC. The mean number of interviews per day was similar for both groups at 10.7 (95% CI 8.7-12.6) for PPDC and 11.8 (95% CI 8.1-15.5) for EDC [39].

A tablet computer-based data collection system was implemented in a large-scale study of trachoma impact assessment surveys in Ethiopia [7]. Data quality outcomes were compared with a similar paper-based survey conducted 7 months earlier in a different part of the country. The sampling units were households (PPDC: 9433 vs EDC: 12,112), and the study enumerated 38,851 individuals in the PPDC survey and 50,858 in the EDC survey. Individuals enumerated with at least 1 blank field in a single respondent response were defined as missing data (1.7% for PPDC vs 1.5% for EDC; P=.01). Missing data at the household level for GPS with blank entries was also reported (EDC: 1.1% vs PPDC: 0.6%; P<.01). Inaccuracy errors were defined only in a percentage of households with an incorrect unique identifying number (PPDC: 2.3% vs EDC: 1.8%). Data entry and analysis were done in less than 1 day for EDC, while it took 14 days for data entry and an additional 5 days for double data entry discrepancy checks for PPDC [7].

Apart from the above studies, interview time or mean data availability duration in the databases were also reported in some papers. A study in Kenya reported faster data upload to a central database in EDC and a median duration for data upload of 7 days (range 1-13 days) after data collection for EDC and 21 days (range 4-56 days) for PPDC (P<.01) [36]. A combined report from Indonesia and the Philippines showed that the median time between data collection and data entry for PPDC surveys was approximately 3 months, compared with 2 days for EDC [34]. Time analysis from a large-scale survey in Tanzania reported that the median duration of an enumeration session per household was 9.4 min (90% central range 6.4-12.2) for paper surveys and 8.3 min (6.1-12.0) for electronic surveys (P=.01) [38].

#### Effect on Cost-effectiveness

Most of the studies reported cost analysis for the expenses incurred to conduct surveys using paper-based and electronic tools. However, the included studies varied significantly in the types and level of cost analysis reported in their groups. Most of the recommendations from the Consolidated Health Economic Evaluation Reporting Standards (CHEERS) statement [47] were not included. In Table 4, we provide basic information on the study and country, analytic method and model, participants per intervention, time horizon, discount rate, currency, included cost inputs, cost ranges, outcomes, consequence, and conclusion information.

**Table 4:** Extracted cost information (based on Consolidated Health Economic Evaluation Reporting Standards evaluation template)

Study and country	Analytic method or model	Interventions studied or population per group (1=PPDC^a^; 2=EDC^b^)	Time horizon, discount rate, currency (base year)	Included cost inputs and assumptions (1=PPDC; 2=EDC)	Data quality outcome link with input cost	Cost range of intervention (1=PPDC; 2=EDC)	Conclusions and remark
King et al [7], Ethiopia	Input cost analysis	9433 12,112	NR^c^ NR US $ (2011)	Printing and data entry costs Tablet and accessories Single-use cost	Not linked	US $13,883 US $10,320	Costs of the equipment in EDC approximately the same as with data entry cost, recurrent use of EDC may save costs. Use of person day for comparison of personnel costs
McLean et al [39], Malawi	Input cost analysis Differential cost	426 558	1 year NR British Pound (2016)	Printing and entry cost and paper archival EDC development and configuration, device cost, data officer cost	Not linked	£18,895 £11,427	In total, the estimated costs for the stages unique to the paper-based process is 65% higher per annum than the unique costs for the EDC system
Giduthuri et al [37], India	Differential input cost analysis	98 interviews for both, and extrapolated for larger samples 1000	NR NR US $ (2013)	Printing and data entry expenses Cost of tablet computers and server charges	Not linked	For 96 interviews, the cost is US $2598 US $2648	The initial investment in tablet-based interviews was higher compared to paper, while the recurring costs per interview were lower with the use of tablets EDC is less expensive for larger surveys
Mukasa et al [38], Tanzania	Cost and cost-effectiveness	1000 households for both	NR (used deflator values) NR (used the number of households) US $ (2008)	All fixed costs and running costs To estimate the costs for 2015, the formula for expenditure in 2016 = Expenditure in 2008 × Deflator 2016, deflator 2008 Deflator values of 209.5 for 2008 and 233.6 for 2016	Error rate	For 1000 households, error-free data set: US $1161 US $9380 Crude data set: US $993 US $891	For error-free data sets, surveys using electronic tools, compared with paper-based tools, were less expensive by 28% for recurrent and 19% for total costs
Zhang et al [42], China	Input cost analysis	60 60 Extrapolated to 1200 1600	NR NR (used projected samples) US $ (2012)	Both EDC and PPDC: Items for preparation, training, fieldwork and data collection, and logistics Only printing and transporting the questionnaire, stationery, and data entry Only renting the smartphone and the software Assumptions for the extrapolation are not known	Not linked	Sample size: 60 each US $1500 US $2700 Sample size: 1200 US $41,570 Cost per sample, US $23.77 Sample size: 1600 US $28,520 Cost per sample, US $25.98	The mean costs per questionnaire were higher for the smartphone questionnaire No linked analysis for data quality and cost
Njuguna et al [36], Kenya	Input cost analysis	Both 880 Extrapolated for the first year’s establishing and second year’s operating cost	NR 2 years US $ (2011, 2012)	First- and second-year costs of starting up and operating based on payroll information	Not linked	First year: US $15,999 US $17,500 Second year: US $54,001 US $50,200	For establishment cost, EDC 9.4% more than PPDC; in 2 years, EDC costs reduced 7% compared to PPDC No linked analysis for data quality and cost
Dillon et al [41], South Africa	Input and economic analysis	95 105	British pound (2012)	Salary per correctly entered question Technology costs and tablet computer and additional overhead costs (storage space for paper hard copies; office space for data entry clerk, and for EDC, hardware maintenance and upkeep) Formulas presented by Walther et al [19]. Assumed minimum staffing: 1 field worker, 1 data entry clerk, and 1 data supervisor 1 field worker and 1 data manager (EDC) Calculation based on 46 questions in a questionnaire, 5 interviews per day, 22 working days per month, 110 interviews per month, and 5060 questions per month	Error rate	Salary cost per month: £1000 £915 Equipment cost: £420 £1036	EDC salary cost per correctly entered question is 0.5 times that of PPDC Initial technology costs for the EDC is 2.47 times that of PPDC Cost per question EDC: £0.18 PPDC: £0.20 EDC saved £101.20 per month EDC cost recoup time 6 months
Flexman et al [34], Bangladesh and Philippines	Input data analysis	5398 516	2013 verbal autopsies’ data collection in demographic and surveillance sites	Differential cost estimation formula excluding similar cost for EDC and PPDC, for example, data collector cost (see the full paper mentioned on page 4) The cost for data entry for a single enterer is the one-time cost of a computer plus the monthly salary multiplied by the number of months required	Not linked	Printing cost per paper questionnaire US $0.246 (Bangladesh) and US $0.774 (Philippines) Cost of a single electronic tablet US $393.78 (Bangladesh) and US $365.76 (Philippines) Cost of a computer for data entry US $984.49 (Bangladesh) and US $1000.00 (Philippines) Monthly salary of data enterer US $384.62 (Bangladesh) and US $326.00 (Philippines) Average number of samples per person per month 107.8 (Bangladesh) and 145.7 (Philippines)	For small-scale surveys, the upfront costs of purchasing electronic tablets was the primary cost, and it had a higher total cost. For large-scale surveys, the costs associated with data entry exceeded the cost of the tablets, so EDC provides a cheaper method of data collection Historical cost data from 2 countries. For projects that require fewer than 150 tablets and collect over 10,000 surveys, the upfront cost of the tablets will likely be substantially less than the cost of data entry
Lietz et al [40], Burkina Faso	Comparative input cost analysis	10,000 households for both	HDSS^d^ and HMS^e^ 2010 and inflated to 2014 values CDA^f^ actual pretesting expenditure in 2014	Fixed costs: personnel (team lead), office, and housekeeping Variable costs: data collectors, supervisors, consumables, transportation, and training (full text in Textbox 1) Fixed and variable costs Financial costs of the standalone (HDSS and HMS) and integrated (CDA) survey approaches were estimated from the perspective of the implementing agency	Not assessed	Cost per household visit HMS + HDSS (PPDC): US $251,641 CDA (EDC): US $206,937 Cost per (10,000 HDSS) EDC: US $21 PPDC: US $25	Cost analysis estimated that the CDA survey would reduce the annual costs of survey implementation by about US $45,000 No link with the data quality outcome

^a^PPDC: pen and paper data collection.

^b^EDC: electronic data collection.

^c^NR: not reported

^d^HDSS: health and demographic surveillance systems.

^e^HMS: Household Morbidity Survey.

^f^CDA: Comprehensive Disease Assessment.

Studies from Ethiopia [7], Malawi [39], India [37], and Bangladesh and Philippines [34] reported a differential input cost unique to paper-based or electronic tools. For paper-based data collection, these were printing and data entry costs, and for EDC systems, the cost of electronic devices’ hardware, software, and accessories. The general assumption was that all other costs, such as personnel costs, were the same for both tools. Another cost assumption was that of the cost of small-scale, short-duration surveys. Such kinds of small costs were extrapolated to large-scale surveys with no clear information about the model or the cost assumptions followed to reach the large-scale costs. The studies concluded that EDC was expensive for small-scale surveys, as the initial investment in hardware and software outweighs the paper-based printing and data entry costs. However, large-scale surveys showed a significant decrease in cost for EDC surveys; for example, the paper-based survey cost was up to 65% higher per annum than the unique costs for the EDC system [39]. None of these studies linked the input cost with data quality errors.

Detailed cost information and the link between cost and data quality were reported in a retrospective data analysis in Tanzania. However, the base year for the cost was 2008, and deflator values of 209.5 for 2008 and 233.6 for 2016 were used to report the costs. For 1000 households, the cost per error-free data set was US $11,610 for PPDC and US $9380 for EDC. For error-free data sets, surveys using electronic tools—compared with paper-based tools—were 28% less expensive in recurrent costs and 19% less expensive in total costs [38].

A study in South Africa also reported cost inputs linked to data quality outcomes [41]. The formula presented by Walther et al [19] was used with minimum staffing: for PPDC, 1 field worker, 1 data entry clerk, and 1 data supervisor, and for EDC, 1 field worker and 1 data manager. In addition, 46 questions in a questionnaire, 5 interviews per day, 22 working days per month, 110 interviews per month, and 5060 questions per month were planned. The EDC salary cost per correctly entered question was 0.5 times that of PPDC. Overall, the cost per question was £0.18 for EDC and £0.20 for PPDC. The equipment cost for PPDC was £420, compared with £1036 for EDC. EDC saved £101.20 per month, and the EDC cost recoup time was reported as 6 months.

Lingani et al [40] in Burkina Faso and Njuguna et al [36] in Kenya reported a detailed financial cost comparison for the establishment of PPDC and EDC at HDSS and a hospital-based surveillance system, respectively. The Kenyan report indicated that during establishment, the cost of EDC was 9.4% higher than that of PPDC. However, after 2 years, EDC costs decreased by 7%, compared with PPDC (see Table 4 for detailed cost information).

#### Technology Characteristics, User Preference, and Acceptance

The mobile devices used for data collection included PDAs, smartphones, tablet computers, and notebooks (Table 5). The included studies (6/12, 50%) also reported the use of open source Android apps called open data kit apps to customize the software according to their needs. Microsoft Windows and BlackBerry operating systems were installed on mobile devices.

Data transfer from mobile devices to the central server was conducted using one of the following methods:

- Direct transfer from the data collection site to the server using a mobile data network [34-36] and secure virtual private network [45].
- Direct transfer using Wi-Fi connection only [37,39].
- Transfer using secure digital memory card [7,38].
- Transfer using USB cable [41].

Data transfer to a server located in a foreign country using a mobile network was not considered appropriate in some studies due to data ownership or security concerns [7,43].

**Table 5:** Extracted intervention or technology characteristics

Study and country	Analytic method or model	Interventions studied or population per group (1=PPDC^a^; 2=EDC^b^)	Time horizon, discount rate, currency (base year)	Included cost inputs and assumptions (1=PPDC; 2=EDC)	Data quality outcome link with input cost
Njuguna et al [36], Ethiopia	HTC smartphone Software: FAST^a^ kit: Microsoft Purchased	Direct to server with mobile network	Programmed checks and restrictions Error message notification for inaccurate data entry	Limited or poor internet network Occasional server communication breakdowns Delayed data submission	Data saved in the smartphone’s memory and later uploaded on to the server from convenient places
Ahmed et al [35], Sudan	Samsung smartphone Customized ODK^b^ Open source	Mobile internet network	ODK functionalities	Limited or poor internet network in certain areas Unexpected software closure or freeze Accessibility of the aggregate website in Sudan was challenging Using Arabic language in the ODK was challenging for form development and data retrieval	Used ODK offline and submitted the data after restarting the smartphone
King et al [7], Ethiopia	Samsung tablet computer Swift Insights Mobile 1.1 Barcode scanner 4.3.1 Open source developed	SD^c^ card with password-protected downloading to the supervisor’s laptop	ODK functionalities+ User-defined survey preferences, generation of unique record identification (Textbox 1)	Limited or poor internet network in certain areas	Data stored first on the supervisor laptop and then uploaded to the local server to maintain the sovereignty and security of the data set
Flexman et al [34], Bangladesh and Philippines	Samsung tablets ODK Customized in ODK	Direct to server with mobile network	ODK functionalities (no explicit description)	Sufficiently strong internet network	Purchase memory cards for the tablets to back up data locally
Giduthuri et al [37], India	Samsung Note external recorder Enhanced ODK development	Encrypted and uploaded over a Wi-Fi connection to a central server after returning to the office	Not described	Required highly trained interviewers Data loss by the interviewer due to accidentally pressing the delete button	Not described
Musaka et al [38], Tanzania	PDA BlackBerry HRS^d^ software BlackBerry OS^e^ Customized in HRS software	Micro SD card	Skip function	Device stopped functioning during interview or submission	Not described
McLean et al [39], Malawi	Toshiba tablets and Samsung smartphones ODK Contractual payment for form development	Secured wireless network (not relayed on phone network)	ODK functionalities	The existing wireless network was sufficiently strong to upload the data Adequate battery life for a day Lack of internal staff for form development Required outsourcing cost 11 of 92 tablets broke in 4 years and had to be replaced Only 1 functioning tablet and 1 smartphone went missing over the 4-year period	A dedicated, secured device charging area
Dillon et al [41], South Africa	Tablet, PC, and mobile phone Self-developed a C#-based program with XML	Data transfer through USB connections, avoiding the need for a constant internet connection	Facilitate data checks and early detection and correction of faulty procedures and data management	A well-run staff training program Staff was already technically trained Lack of reliable internet connection	Data transfer through USB
Jing et al [43], China	Smartphone ODK and importing and extraction software ODK Briefcase v 1.4 Production	Stored safely on the smartphone and uploaded to a computer secured by a password. Compiled data sent to the country coordinator	ODK functionalities	Interviewers do not have direct access to submit the data to the server in Washington, forcing them to store the data on the smartphones for a short period Loss of data due to smartphone damage Uploading data was a challenge for older smartphone users	Building a server in China that can be easily accessed would facilitate improved data security and immediate assignment of cause of death on smartphones at the time of interview
Byass et al [44], Burkina Faso	PDA Pendragon 4 software programmed	Data copied for PDAs’ memory cards	GPS, time stamp	Use several available mobile-charging arrangements in vehicles or solar panels Temporary system clock changes in a few PDAs Some incomplete GPS data strings 48/151 PDA encountered technical errors	Saving data from the PDAs’ internal volatile memory to nonvolatile memory cards. Protective plastic cover for the safety of PDA was important
Maduga et al [46], Nigeria	Mobile phone ODK Android Locally customized	Trained to upload completed forms onto a secure server, with back-end access provided to only the research team lead	ODK functionalities	Limited network connection Fluctuation of power and internet connection	The extra 2 phones served as backup in the event of malfunction or challenges with the global system for mobile communication mobile and data networks. Multiple SIM cards were provided in an attempt to mitigate the problem

^a^FAST: Field Adapted Survey Toolkit.

^b^ODK: Open Data Kit.

^c^SD: Secure Digital.

^d^HRS: Household Registration System.

^e^OS: operating system.

#### Technical Challenges in Electronic Data Collection

Limited or poor internet connectivity, occasional server communication interruptions, language challenges, the need for highly trained data collectors, device stack or freezing, device loss and breakdowns, and limited battery or power sources are among the technological challenges faced in the implementation of EDC systems (Table 5). The solutions include offline storage and transfer as soon as the data collectors obtain reliable internet connection (store and forward methods), transferring data using USB cables or secure digital cards, purchasing backup mobile devices or batteries, and using paper questionnaires at times of device malfunction.

#### Preference, Acceptability, and Feelings

A total of 6 papers [7,34,37-39,42] reported that user preferences, acceptance, and opinions were assessed using formal evaluation methods, such as individual or focus groups or qualitative interviews. Detailed comparative advantages and disadvantages of paper and electronics tools are reported in thematic-based tables in [7,38,39].

The use of smartphones to collect data was faster, easier to follow, and more convenient, as the data collectors did not have to carry cumbersome paper questionnaires and less space was needed to store their data collection tools. Additional functionalities—such as automatic retrieval of respondents and other members of the household or GPS functionalities—are also reported as an advantage.

The risk of data loss with paper-based surveys was perceived as being less than that with EDC, as paper questionnaires are tangible and enable immediate review, identification, and correction of mistakes. Paper surveys were also perceived to be easier for manipulating, adding, or changing data—for instance, including a household member absent during the survey who was later encountered by the survey team. The automated skip function was advantageous and time saving. The enumerators did not have to read the questions on every visit to the same household.

Enumerators described that the devices felt exciting, interesting, and prestigious, and they were skilled professionals in the eyes of the community. Some fieldworkers felt that EDC interviews took longer, and occasionally, devices froze during an interview.

Some studies that were excluded from our review also offer important insights about preference, acceptability, and local experiences [48-51].

## Discussion

This systematic review synthesized the available comparative evidence on paper-based and electronic data collection tools and the potential effect of using these tools on data quality, implementation cost, and user preferences in interview-administered public health surveys. The systematic review included studies from 2008 to 2018 that were identified through multiple online electronic database searches. We identified more than 3500 papers and screened more than 2500 titles and abstracts to include 14 full-text papers based on our inclusion criteria. We extracted and synthesized available evidence regarding data quality, cost-effectiveness, timeliness, and user preferences. No paper has reported a study design with a classical randomized control approach. Randomization was reported to indicate either respondent allocation to paper-based or electronic tools or data collectors’ exposure to one of the data collection tools. Meta-analysis was not possible due to the heterogeneous nature of study designs, measurements and outcome types, and study settings. Instead, a narrative synthesis based on predefined data quality, cost and related outcomes’ acceptability, and preferences was conducted.

We employed a rigorous systematic review process to formulate the research questions, prepare individual database-tailored search strategies, execute searches in multiple databases, and independently screen and extract evidence from thousands of papers.

However, the results were inadequate for meta-analysis. The final included studies were heterogeneous and could not be combined to generate better estimates. Low-quantity and low-quality phenomena are becoming evident in most recent systematic reviews assessing mHealth or eHealth outcomes [13,29,30].

This scarcity might result from the following reasons: lack of primary studies with a rigorous study design, insufficient search strategy or review process, or unnecessary narrowing of a study focus. The most commonly reported reason is a lack of sufficient, well-planned, rigorous studies. The lack of evidence might be due to reluctance to evaluate the system after implementation and due to publication bias because of unsuccessful or disappointing findings [52]. Apart from a few studies [35,37,39,41,42], those included in our systematic review were not primarily designed to evaluate the impact of EDC compared with PPDC. Instead, the studies were a byproduct of survey experience from a comparative analysis using their secondary data. Information from such implementation practices can provide insights or lessons for readers, but the comparative outcomes might be influenced by unplanned and uncontrolled confounding variables [7,36]. Elimination of observed and unobserved factors that might otherwise plausibly explain the difference in outcomes in the study design can increase confidence in the assertion that the estimated impact constitutes the real impact of the tools. The studies included in this review suffered from multiple biases during sample size estimation, selection (purposive vs random selection), and data quality outcome measurement level (before or after data entry). We recommend that future research focus specifically on the mode of data collection measurement and on quantifying the impacts with sound research designs.

Generating a full economic evaluation of the evidence facilitates a comparison between interventions in terms of their costs and intended outcomes and can be used to inform decisionmakers or funders of the available choices among alternatives upon cost justification [53,54]. In our systematic review, we attempted to extract the available cost information using the CHEERS checklist [47]; however, most of the expected items in the checklist were not reported. A majority of the studies lacked a detailed description of unit costs, data sources, and cost calculations. Moreover, most used a time horizon of 1 year and failed to assess long-term costs and data quality effects. The rationale for the choice of the time horizon was also not explicitly stated.

Despite these limitations, the available cost data could provide clues regarding the existing cost parameters for paper- and electronic data collection systems. Two studies managed to link the cost of implementing EDC and PPDC tools to data quality. Such cost-effectiveness analyses should be encouraged in future studies. There is no clear answer or guideline to shape the type and level of rigorous studies in health information technology evaluation [55,56]. Details of evidence-based health informatics history, current practices, and future recommendations are discussed elsewhere [52]. Further debate and consensus among academics and researchers in biomedical informatics should continue to determine how and when health information technology evaluation is rigorous and produces good quality data [57].

The studies identified in this review were conducted in various countries and in the context of different health care systems. Generalizing and applying results from different contexts is difficult because of variations in clinical practice, costs, and their analysis. However, what was consistent across all studies was a lack of reporting on the feasibility of adopting these technologies based on economic and organizational factors.

It was surprising to see limited publications from global survey implementation organizations for the DHS and INDEPTH network groups, as those projects have many years of multinational implementation experience [4,58]; however, apart from experience reports, comparative evaluation studies in those areas are rare. Further evaluation research in these projects might produce evidence of data quality, cost, timeliness, and the success and failure factors for multinational projects.

There are positive perceptions regarding the acceptability, usability, and preference of EDC over PPDC among data collectors. This positivity is because technology enables data collectors to focus on their work, get immediate feedback regarding their mistakes, correct their errors while in the field, and leave few data quality issues to revisit. It is not known whether this excitement is a short-term effect immediately after the technology introduction or a stable long-term view based on longer exposure. A short period of technophilic or technophobic attitudes might lead to inaccurate overall impression of the tool, as accurate impression can only develop with longer exposure to the technology [59]. The generalizability and applicability of the results, given the different types of devices with different technical specifications and the rapid pace at which technology advances, need critical evaluation. The generalizability of the findings of this systematic review is also challenged by the studies themselves, considering the variations in the characteristics of data collectors, level of outcome measurements, settings of the survey, and the psychometric properties of the survey questionnaires. 

### Conclusions

This systematic review showed that, despite consistent claims of a positive impact of technology on data quality and cost-effectiveness, the available evidence is small in quantity and low in quality. Purposefully designed comparative studies assessing the impact of data quality and cost-effectiveness are needed for implementation in organizations and by decision makers.

Despite the heterogeneity and low quality of the included studies, their qualitative synthesis showed the superiority of EDC systems over paper-based systems for data quality, process efficiencies, and cost.

Comparative evaluation studies sourced from international survey-implementing organizations where their routine data collection mode is EDC can provide a better platform for impact evaluation research in large-scale surveys.

## Appendix

Multimedia Appendix 1 Additional search strategies.

## Abbreviations

CHEERS: Consolidated Health Economic Evaluation Reporting Standards
CRF: clinical case report form
DHS: demographic and health survey
eCRF: electronic clinical report form
HDSS: health and demographic surveillance system
INDEPTH: International Network for the Demographic Evaluation of Populations and Their Health
mHealth: mobile health
RR: risk ratio
