# Supplementary material for: Data Quality and Cost-effectiveness Analyses of Electronic and Paper-Based Interviewer-Administered Public Health Surveys: Systematic Review
Source: J Med Internet Res. 2021 Jan 22;23(1):e21382. doi: 10.2196/21382 (PMC7864777; doi:10.2196/21382)
Supplement: Multimedia Appendix 1 [file jmir_v23i1e21382_app1.docx]

**MEDLINE(R) and Epub Ahead of Print, In-Process & Other Non-Indexed Citations, Daily and Versions(R) via Ovid**

1. (((tablet or handheld or hand held or electronic) adj2 (device* or computer*)) or ((electronic or digital) adj2 (form? or data capture* or survey* or case report form? or data collection?)) or Open Data Kit or ODK or EDC or eCRF or eHealth or mHealth or digital health or Android or tablet? or PDA? or personal digital assistant? or app? or (mobile adj2 (technolog* or application? or app?)) or ((mobile or cell* or smart) adj2 phone*) or smartphone* or cellphone*).ti,ab.

2. exp "mobile applications"/

3. exp "computers, handheld"/

4. exp "electronic health records"/

5. exp "cell phone"/

6. or/2-5

7. 1 or 6

8. (field work or fieldwork or HDSS or CAPI or computer assisted personal interviewing or questionnaire* or survey* or interview* or (population adj2 surveillance) or DHS or EDC or (data adj2 (gather* or captur*)) or (health and demographic surveillance system?)).ti,ab.

9. exp "surveys and questionnaires"/

10. exp "interviews as topic"/

11. or/9-10

12. 8 or 11

13. ((cost? adj2 (analy?s or comparison* or saving? or measure? or effectiv* or reduction? or reduce? or reduction or reducing or decrease? or decreasing)) or (cost benefit adj2 (analy?s or comparison* or measure?)) or (cost utility adj2 (analy?s or comparison* or measure?)) or economic evaluation? or quality control? or (data adj2 (quality or accuracy or accurate* or error? or error rate? or incomplete* or complete* or inaccurate* or inaccuracy or valid*))).ti,ab.

14. exp "costs and cost analysis"/

15. exp "data accuracy"/

16. or/14-15

17. 13 or 16

18. 7 and 12 and 17

19. limit 18 to yr="2008 -Current"

**PsycINFO via Ovid**

1. (((tablet or handheld or hand held or electronic) adj2 (device* or computer*)) or ((electronic or digital) adj2 (form? or data capture* or survey* or case report form? or data collection?)) or Open Data Kit or ODK or EDC or eCRF or eHealth or mHealth or digital health or Android or tablet? or PDA? or personal digital assistant? or app? or (mobile adj2 (technolog* or application? or app?)) or ((mobile or cell* or smart) adj2 phone*) or smartphone* or cellphone*).ti,ab.

2. exp "mobile devices"/

3. exp "computer peripheral devices"/

4. exp "cellular phones"/

5. or/2-4

6. 1 or 5

7. (field work or fieldwork or HDSS or CAPI or computer assisted personal interviewing or questionnaire* or survey* or interview* or (population adj2 surveillance) or DHS or EDC or (data adj2 (gather* or captur*)) or (health and demographic surveillance system?)).ti,ab.

8. exp "surveys"/

9. exp "questionnaires"/

10. exp "interviews"/

11. or/8-10

12. 7 or 11

13. ((cost? adj2 (analy?s or comparison* or saving? or measure? or effectiv* or reduction? or reduce? or reduction or reducing or decrease? or decreasing)) or (cost benefit adj2 (analy?s or comparison* or measure?)) or (cost utility adj2 (analy?s or comparison* or measure?)) or economic evaluation? or quality control? or (data adj2 (quality or accuracy or accurate* or error? or error rate? or incomplete* or complete* or inaccurate* or inaccuracy or valid*))).ti,ab.

14. exp "costs and cost analysis"/

15. 13 or 14

16. 6 and 12 and 15

17. limit 16 to yr="2008 -Current"

**CINAHL via EBSCO**

S23 S9 AND S16 AND S21
Limiters - Published Date: 20080101-20181231

S22 S9 AND S16 AND S21

S21 S19 OR S20

S20 (MH "Costs and Cost Analysis+")

S19 S17 OR S18

S18 AB ((cost? n2 (analy?s or comparison* or saving? or measure? or effectiv* or reduction? or reduce? or reduction or reducing or decrease? or decreasing)) or (cost benefit n2 (analy?s or comparison* or measure?)) or (cost utility adj2 (analy?s or comparison* or measure?)) or economic evaluation? or quality control? or (data adj2 (quality or accuracy or accurate* or error? or error rate? or incomplete* or complete* or inaccurate* or inaccuracy or valid*)))

S17 TI ((cost? n2 (analy?s or comparison* or saving? or measure? or effectiv* or reduction? or reduce? or reduction or reducing or decrease? or decreasing)) or (cost benefit n2 (analy?s or comparison* or measure?)) or (cost utility adj2 (analy?s or comparison* or measure?)) or economic evaluation? or quality control? or (data adj2 (quality or accuracy or accurate* or error? or error rate? or incomplete* or complete* or inaccurate* or inaccuracy or valid*)))

S16 S12 OR S15

S15 S13 OR S14

S14 (MH "Questionnaires+")

S13 (MH "Data Collection Methods+")

S12 S10 OR S11

S11 AB (field work or fieldwork or HDSS or CAPI or computer assisted personal interviewing or questionnaire* or survey* or interview* or (population n2 surveillance) or DHS or EDC or (data n2 (gather* or captur*)) or (health and demographic surveillance system?))

S10 TI (field work or fieldwork or HDSS or CAPI or computer assisted personal interviewing or questionnaire* or survey* or interview* or (population n2 surveillance) or DHS or EDC or (data n2 (gather* or captur*)) or (health and demographic surveillance system?))

S9 S3 OR S8

S8 S4 OR S5 OR S6 OR S7

S7 (MH "Cellular Phone+")

S6 (MH "electronic health records+")

S5 (MH "mobile applications+")

S4 (MH "Computers, Hand-Held+")

S3 S1 OR S2

S2 AB ( (((tablet or handheld or hand held or electronic) n2 (device* or computer*)) or ((electronic or digital) n2 (form? or data capture* or survey* or case report form? or data collection?)) or Open Data Kit or ODK or EDC or eCRF or eHealth or mHealth or digital health or Android or tablet? or PDA? or personal digital assistant? or app? or (mobile n2 (technolog* or application? or app?)) or ((mobile or cell* or smart) n2 phone*) or smartphone* or cellphone*) )

S1 TI ( (((tablet or handheld or hand held or electronic) n2 (device* or computer*)) or ((electronic or digital) n2 (form? or data capture* or survey* or case report form? or data collection?)) or Open Data Kit or ODK or EDC or eCRF or eHealth or mHealth or digital health or Android or tablet? or PDA? or personal digital assistant? or app? or (mobile n2 (technolog* or application? or app?)) or ((mobile or cell* or smart) n2 phone*) or smartphone* or cellphone*) )

**EconLit with Full Text via EBSCO**

S11 S3 AND S6 AND S9
Limiters - Published Date: 20080101-20191231

S10 S3 AND S6 AND S9

S9 S7 OR S8

S8 AB ((cost* n2 (analy?s or comparison* or saving* or measure* or effectiv* or reduction* or reduce* or reducing or decrease? or decreasing)) or ("cost benefit" n2 (analy?s or comparison* or measure*)) or ("cost utility" n2 (analy?s or comparison* or measure*)) or "economic evaluation*" or "quality control*" or (data n2 (quality or accuracy or accurate* or error* or "error rate*" or incomplete* or complete* or inaccurate* or inaccuracy or valid*)))

S7 TI ((cost* n2 (analy?s or comparison* or saving* or measure* or effectiv* or reduction* or reduce* or reducing or decrease? or decreasing)) or ("cost benefit" n2 (analy?s or comparison* or measure*)) or ("cost utility" n2 (analy?s or comparison* or measure*)) or "economic evaluation*" or "quality control*" or (data n2 (quality or accuracy or accurate* or error* or "error rate*" or incomplete* or complete* or inaccurate* or inaccuracy or valid*)))

S6 S4 OR S5

S5 AB ("field work" or fieldwork or HDSS or CAPI or "computer assisted personal interviewing" or questionnaire* or survey* or interview* or (population n2 surveillance) or DHS or EDC or (data n2 (gather* or captur*)) or "health and demographic surveillance system*")

S4 TI ("field work" or fieldwork or HDSS or CAPI or "computer assisted personal interviewing" or questionnaire* or survey* or interview* or (population n2 surveillance) or DHS or EDC or (data n2 (gather* or captur*)) or "health and demographic surveillance system*")

S3 S1 OR S2

S2 AB (((tablet or handheld or "hand held" or electronic) n2 (device* or computer*)) or ((electronic or digital) n2 (form* or "data capture*" or survey* or "case report form*" or "data collection*")) or "Open Data Kit" or ODK or EDC or eCRF or eHealth or mHealth or "digital health" or Android or tablet* or PDA* or "personal digital assistant*" or app or apps or (mobile n2 (technolog* or application* or app or apps)) or ((mobile or cell* or smart) n2 phone*) or smartphone* or cellphone*))

S1 TI (((tablet or handheld or "hand held" or electronic) n2 (device* or computer*)) or ((electronic or digital) n2 (form* or "data capture*" or survey* or "case report form*" or "data collection*")) or "Open Data Kit" or ODK or EDC or eCRF or eHealth or mHealth or "digital health" or Android or tablet* or PDA* or "personal digital assistant*" or app or apps or (mobile n2 (technolog* or application* or app or apps)) or ((mobile or cell* or smart) n2 phone*) or smartphone* or cellphone*))

**Cochrane Database of Systematic Reviews and Cochrane Central Register of Trials (CENTRAL) via Cochrane Libraries**

1. (((tablet or handheld or "hand held" or electronic) near/2 (device* or computer*)) or ((electronic or digital) near/2 (form? or "data capture" or survey* or "case report form" or "case report forms" or "data collection" or "data collections")) or "Open Data Kit" or ODK or EDC or eCRF or eHealth or mHealth or "digital health" or Android or tablet? or PDA? or "personal digital assistant" or "personal digital assistants" or app? or (mobile near/2 (technolog* or application? or app?)) or ((mobile or cell* or smart) near/2 phone*) or smartphone* or cellphone*):ti,ab

2. MeSH descriptor: [Mobile Applications] explode all trees

3. MeSH descriptor: [Computers, Handheld] explode all trees

4. MeSH descriptor: [Electronic Health Records] explode all trees

5. MeSH descriptor: [Cell Phone] explode all trees

6. {OR #1-#5}

7. #1 OR #6

8. ("field work" or fieldwork or HDSS or CAPI or "computer assisted personal interviewing" or questionnaire* or survey* or interview* or (population near/2 surveillance) or DHS or EDC or (data near/2 (gather* or captur*)) or "health and demographic surveillance system" or "health and demographic surveillance systems"):ti,ab

9. MeSH descriptor: [Surveys and Questionnaires] explode all trees

10. MeSH descriptor: [Interviews as Topic] explode all trees

11. #9 OR #10

12. #8 OR #11

13. (cost? near/2 (analy?s or comparison* or saving? or measure? or effectiv* or reduction? or reduce? or reduction or reducing or decrease? or decreasing)) or ("cost benefit" or "cost utility") near/2 (analy?s or comparison* or measure?) or "economic evaluation?" or "quality control?" or (data near/2 (quality or accuracy or accurate* or error? or "error rate" or "error rates" or incomplete* or complete* or inaccurate* or inaccuracy or valid*)):ti,ab

14. MeSH descriptor: [Costs and Cost Analysis] explode all trees

15. MeSH descriptor: [Data Accuracy] explode all trees

16. #14 OR #15

17. #13 OR #16

18. #7 AND #12 and #17

19. #7 AND #12 and #17
with Cochrane Library publication date from Jan 2008 to Dec 2019

**Social Science Citation Index Expanded and Science Citation Index Expanded via Web of Science**

#11 #9 AND #6 AND #3

Refined by: PUBLICATION YEARS: (2019 OR 2011 OR 2018 OR 2010 OR 2017 OR 2009 OR 2016 OR 2008 OR 2015 OR 2014 OR 2013 OR 2012)

#10 #9 AND #6 AND #3

#9 #8 OR #7

#8 AB=((cost$ near/2 (analy?s or comparison* or saving$ or measure$ or effectiv* or reduction$ or reduce$ or reduction or reducing or decrease$ or decreasing)) or ("cost benefit" near/2 (analy?s or comparison* or measure$)) or ("cost utility" near/2 (analy?s or comparison* or measure$)) or "economic evaluation$" or "quality control$" or (data near/2 (quality or accuracy or accurate* or error$ or "error rate$" or incomplete* or complete* or inaccurate* or inaccuracy or valid*)))

#7 TI=((cost$ near/2 (analy?s or comparison* or saving$ or measure$ or effectiv* or reduction$ or reduce$ or reduction or reducing or decrease$ or decreasing)) or ("cost benefit" near/2 (analy?s or comparison* or measure$)) or ("cost utility" near/2 (analy?s or comparison* or measure$)) or "economic evaluation$" or "quality control$" or (data near/2 (quality or accuracy or accurate* or error$ or "error rate$" or incomplete* or complete* or inaccurate* or inaccuracy or valid*)))

#6 #5 OR #4

#5 AB=("field work" or fieldwork or HDSS or CAPI or "computer assisted personal interviewing" or questionnaire* or survey* or interview* or (population near/2 surveillance) or DHS or EDC or (data near/2 (gather* or captur*)) or "health and demographic surveillance system?")

#4 TI=("field work" or fieldwork or HDSS or CAPI or "computer assisted personal interviewing" or questionnaire* or survey* or interview* or (population near/2 surveillance) or DHS or EDC or (data near/2 (gather* or captur*)) or "health and demographic surveillance system?")

#3 #2 OR #1

#2 AB=(((tablet or handheld or "hand held" or electronic) NEAR/2 (device* or computer*)) or ((electronic or digital) NEAR/2 (form$ or "data capture*" or survey* or "case report form$" or "data collection$")) or "Open Data Kit" or ODK or EDC or eCRF or eHealth or mHealth or digital health or Android or tablet$ or PDA$ or "personal digital assistant$" or app$ or (mobile NEAR/2 (technolog* or application$ or app$)) or ((mobile or cell* or smart) NEAR/2 phone*) or smartphone* or cellphone*)

#1 TI=(((tablet or handheld or "hand held" or electronic) NEAR/2 (device* or computer*)) or ((electronic or digital) NEAR/2 (form$ or "data capture*" or survey* or "case report form$" or "data collection$")) or "Open Data Kit" or ODK or EDC or eCRF or eHealth or mHealth or digital health or Android or tablet$ or PDA$ or "personal digital assistant$" or app$ or (mobile NEAR/2 (technolog* or application$ or app$)) or ((mobile or cell* or smart) NEAR/2 phone*) or smartphone* or cellphone*)

|  |
| --- |
